# Developmental Disability: Families and Functioning in Child and Adolescence

**DOI:** 10.3389/fresc.2021.709984

**Published:** 2021-08-23

**Authors:** Peter Rosenbaum

**Affiliations:** CanChild Centre, Department of Pediatrics, McMaster University, Hamilton, ON, Canada

**Keywords:** childhood disability, ICF, F-words, family well-being, family-centered service

## Abstract

The WHO's International Classification of Functioning, Disability and Health (ICF) provides an integrated framework for health for everyone. Several aspects of this approach to health allow us to see people's lives in a richer and more holistic manner than has traditionally been the case based on diagnosis alone. These features include the positive language (emphasizing in particular “activity,” “participation,” and “personal factors”); the interconnections of the parts of this “dynamic system,” in which every component can influence every other one; and the formal inclusion of “contextual factors”—personal and environmental—that are otherwise too easy to take for granted and then ignore. This paper addresses the “environmental” dimension of the ICF framework—specifically referring to “family” as the central environmental force in the lives of children and adolescents. The perspectives of the author are those of a developmental pediatrician, whose career has focused on children with conditions that challenge their development, and their families. Lessons learned from a lifetime of work—including teaching and research as well as clinical services—are offered. Particular emphases will be on (i) the importance of focusing on the family in a non-judgmental “family-centered” way; (ii) how conceptual ideas about child (and family) development and parenting are as important as technical approaches to intervention; and (iii) how the ICF framework “allows”—indeed encourages—such a focus to have value and importance equal to the best of biomedical interventions. Examples from current research will illustrate how these ideas can be implemented.

## Introduction

### The Author's Perspective

This paper is not a conventional literature review, but rather a personal essay about a topic in which the author has longstanding interest and engagement. It draws on my perspectives and experiences as a developmental pediatrician—a clinician, teacher, and health services researcher in childhood disability. The ideas offered here are predicated on two statements of the obvious. First, in the field of child health, we work with families. Every child we ever see comes wrapped in a family. By “family” I refer to one or more responsible adults who have a central on-going caregiving role to protect and nurture that child. In the current era, families appear in a variety of constellations. Whatever their make-up, however, they must be the unit of our clinical attention. That means that we cannot consider only the child without an equal interest in, and focus on, their “family.” Second, if it is true that “It takes a village to raise a child,” it is equally true that a child's challenges of health or development affect the whole family (and beyond, as I shall argue). This means that the child's issues cannot be addressed solely as an individual's predicament.

### Modern Thinking About Health

**Figure 1:**
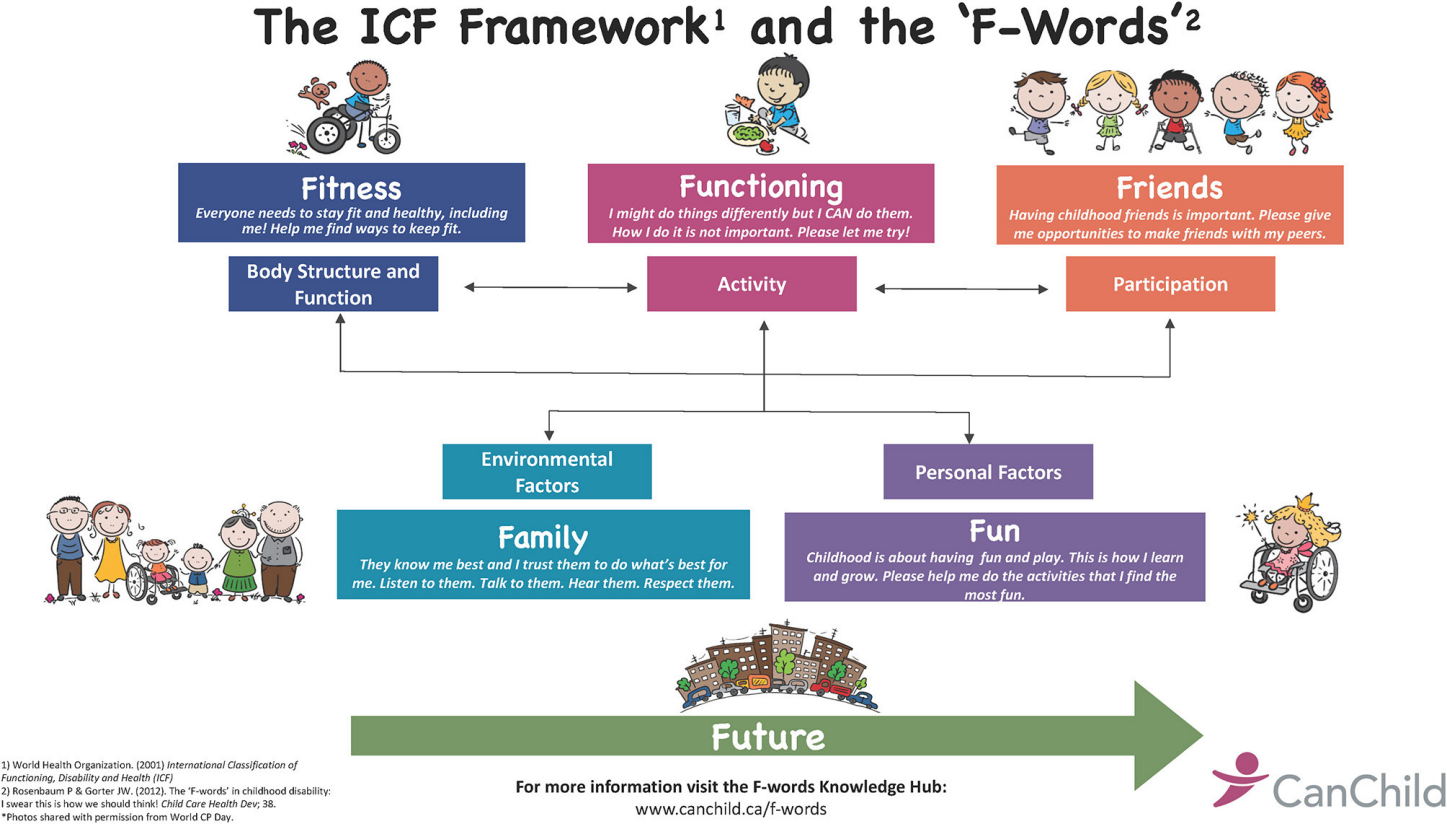
The ICF framework and the F-words.

The centrality of the family in children's lives is not simply the author's personal view. The World Health Organization's 2001 framework for health was formulated within its InternationaI Classification of Functioning, Disability and Health (1) (the ICF). This framework formally identifies “contextual” factors that include “environment.” The nuclear environment of the child is their family. In its efforts to bring the ICF framework to life, CanChild researchers wrote a whimsical paper about “The F-words for Child Development” (2) (see Figure 1), in which the “f-word” family is used to illustrate the “environmental factors” component of the ICF.

What are the implications of this interest in family? I believe that all our considerations of the health, development and well-being of children and youth need to be built around a set of values, namely: (i) an understanding of each specific family, with its strengths, resources and challenges; (ii) acceptance of each family's individuality and culture, within which, as service providers, we need to operate; and (iii) the importance of a working relationship with families based on a shared, mutually respectful partnership. The famous physician and educator Sir William Osler offered the opinion that: “The good physician treats the disease; the great physician treats the patient who has the disease.” (3). In our field, one might rephrase this idea and state: “The good child health professional treats the child's condition; the great child health professional treats the family of the child who has the condition.”

### How Can We Think About Our Work With Families?

In reflecting on how health professionals can work most effectively with families of children and youth with chronic conditions, we will explore both the *processes* by which we should engage with families, including consideration of the values that underpin our interactions, and the *content* and nature of our advice and counsel. Both the processes and content of our work with families continue to evolve, as will be outlined briefly in the respective sections of this paper.

## What Do We Mean by the *Processes* of Working With Families of Children and Youth?

### Family-Centered Service

The concept of “family-centered care” emerged in the US in the 1960s, promoted by parents of children who required hospitalization, and whose engagement with their child's care in hospital was very limited. In fact, in the later 1960s when I trained at a major children's hospital, there were *visiting hours for parents*! One hopes that in the twenty-first century this strange situation is a historical anomaly, and no longer a reality anywhere.

What do we mean by the term “family-centered service” or FCS? (We prefer the term “service” to “care,” as professionals are “service providers.”) We see FCS as “…a set of values, attitudes and approaches to service for children with special needs and their families. The family works together with service providers to make informed decisions about the services and supports the child and family receive. In FCS, the strengths and needs of all family members are considered.” (4) FCS is built on three premises: (i) parents/caregivers are the experts on their child's needs and abilities; (ii) each family is unique; and (iii) the family is the constant in the child's life.

### How Does FCS Work? How Do We Do It?

If one accepts the premises outlined above, it becomes apparent that we need to work in a mutually respectful partnership with families. As the world's experts on their child (they know them best), parents/caregivers must be involved in decision-making about issues that concern their child and family, and should have ultimate responsibility for the care of their children. The uniqueness and individuality of every family means that we cannot assume anything about their values or beliefs based on ethnicity, religion, skin color or any other external characteristic. Rather, it is essential to understand their predicament as they experience it. A wise parent expressed this idea very clearly when she told me: “You have textbooks; we have story books!” (5). In other words, to provide the optimal services for children and their families we need each other's special insights—service providers' understanding of the clinical realities of diagnosis, prognosis, effective interventions for these conditions, and so on, and families' stated values, beliefs, hopes, expectations, resources, and so on.

### Yes, but What About…?

FCS concepts and practices have taken root in many settings, but there remain people who may be uncomfortable with the idea of FCS. They wonder whether we are now simply expected to do a family's bidding, regardless of our own judgment; or whether we have to wait for parents to decide that they want and need before we can act. They are asking, in effect, “Is the tail wagging the dog?” They also wonder whether FCS actually matters. There are good answers to both these concerns.

Needless to say, we need to understand families' requests and wishes, as a basis for discussion and shared problem-solving. This can happen when we build mutually respectful relationships with families, whereby everything can be discussed and addressed thoughtfully. Our perspectives and interpretations of parents' concerns can be the foundation for conversations about the deeper meaning of a parent's wishes, and possible approaches to address them. As an example, imagine a young child who cannot speak clearly, for whom the parent's main goal is for the child to talk. We can reframe the situation to help parents see that what they want is to understand what's on their child's mind—in which case alternate modes of communication may be an excellent approach while the child continues to develop speech (6).

Here is another strategy that can be effective: when parents are uncertain how best to proceed, we can lay out the range of approaches that other families in this predicament have considered. By providing families an understanding of their options, and listening carefully to their questions, we may be able to “precipitate” a course of action for them based on what they ask us, and on their ideas. Using their words, we may be able to say “It sounds to me like following course of action would make most sense to you at this point.” For example, parents may be uncertain as to whether their child with toe-walking associated with spasticity in calf muscles should have any intervention, and if so, might this be a brace or botulinum toxin injections? Parents seeking long-term changes may find the bracing option more palatable than repeated injections, whereas parents wanting to know if changes in muscle activity improve gait are more likely to opt for a trial of botulinum toxin. This approach has, on many occasions, helped me in my work with families—an example of how we can help them help us help them!

Another less appreciated aspect of being interested in families is to understand what the grandparents know and think (7, 8). We most often meet “parents”—the people who bring their children to us with their concerns. We can forget that most (young) parents have their own living parents—and are therefore also someone's “child!” The people we are seeing as parents are thus living in a “generational sandwich,” and their parents may “suffer twice”—for their grandchild and for their (adult) child's predicament. This reality opens up opportunities for us to ask about and try to understand the roles and influences of grandparents in the lives of the parents and children with whom we are working. Offering parents the opportunity to invite the grandparents to attend clinic visits with them is a powerful and very rewarding way to be family-centered.

It is a statement of the obvious that grandparents are a generation older that their (adult) children. Thus, their perspectives, beliefs, and attitudes about disability may be dated relative to, for example, current ICF-based thinking. Being able both to hear and explore their questions and fears, and share ideas such as those offered here, enables them to at least consider their views in light of concepts and evidence that they otherwise had no reason to know. Although I have never explored this approach systematically, I have done this scores of times and recall these experiences clearly as among the most memorable of my clinical career (I will add that many parents, when offered this opportunity to engage their parents, are convinced that the grandparents will not be interested—only to have the grandparents seize the opportunity and engage actively.)

We have argued that “Parenting is a dance led by the ever-changing child” (9). I believe that this metaphor also applies to our relationships with families. Parents develop and change, become better informed with time and experience, and see their changing child's abilities and needs emerging. When we have been able to create relationships that are built on trust, respect and confidence in each other, each of us can help the other succeed in our shared “dance” toward goals for their child and family.

### Yes, but Does FCS Matter?…

As for the value of FCS to families, there is good evidence of important relationships between parents' reports of their experiences of FCS, using validated tools created with parents for parents (10) and parents' reports of their satisfaction with services, their mental health, and the stress they experience in working with us (11).

In summary, FCS can be learned and practiced, and it does make a difference to parents and families. FCS does “work” (12).

## In What Ways Does the *Content* of Our Advice Matter to Parents and Families?

### What Do They Know Already?

It is important to remember that as specialist service providers we rarely if ever are a family's first-contact experience. That means that it is essential to understand what families “know” already. It is on that foundation of facts, beliefs, information, misconceptions, expectations and values that we will build our relationships with families, as outlined above, and add our advice and ideas to the mix of what is already “there.”

After introductions, and my routine opening question to all parents—“What do you want to boast about concerning your child?”—we need to understand what they have been told. An open-ended question such as “Tell me about your child” or “Tell me how I can be helpful?” allows parents to tell their story in their own words before we bombard them with questions. We want to listen for and learn: Has there been a diagnosis given? What was the tone of the conversations that preceded the referral to us? What have parents been told we (service providers) can/will do? An additional good way to find out both what parents are focusing on and what they have learned is to ask what websites they have explored.

I am especially interested in the idea of what I call the “catalog of doom”—the long list of things that parents have been told their child will *not* be able to do. Conversations with families in which people present a bleak outlook can be devastating to the parents, and are often ill-informed. They may be offered with good intentions, because of a belief that preparing people for the worse is appropriate. My own approach to this “catalog” catastrophizing is to remind parents of three ideas: first, we are rarely as accurate at predicting as we would wish to be; second, children are constantly changing and developing, so an early effort to understand the “story” and its outcome should be avoided, because the plot will continue to unfold; and third, I tell parents that the children rarely “listen” to these bleak prognostications, and often achieve much more than was originally predicted! [A thoughtful exploration of this complex issue of communicating “bad news” is offered by Siegler (13).]

### What Are We Offering in Our Counseling and Advice?

Building on the details that parents have reported in our initial conversations described above, and the specific questions they have for us, we need to address their issues as clearly, specifically, and honestly as possible. Parents may have been told that our services will offer “therapies,” with a more or less explicit implication that these will fix their child's issues. Parents have often read about specific therapies—be they chemical (e.g., botulinum toxin injections), physical (e.g., physical, occupational, and speech-language therapies), technical (e.g., bracing or special Velcro outfits), electronic (e.g., muscle stimulation treatment), etc.—that are reported to be exactly what their child needs. Some of this advice may be relevant to their situation, while other elements may reflect advertisements for what are often referred to as “complementary and alternative therapies” of uncertain value (14, 15). At the same time, if we are honest, we have to acknowledge that much of our accepted conventional intervention is also of uncertain value much of the time.

The reality is that we almost always recommend “programs” of intervention with a mix of ideas and advice, and rarely offer just individual “treatments.” This is because we are trying to promote functioning and development, and not simply to address a specific impairment as one might do in rehabilitating and strengthening weakened thigh muscles after a broken leg.

### What Can We Do Differently?

For these reasons, I believe that we should provide both a context for, and an interpretation of, parents' concerns, and offer a modern view of our thinking. With colleagues in Australia, our CanChild team is involved in a research study to assess the impact of the introduction of a specific programmatic set of ideas we believe are important for parents. These five interactive workshops present (i) WHO's ICF ideas about health and the F-words framework; (ii) Ideas about child, family and sibling development; (iii) “Parenting is a dance led by the child;” (iv) Strategies for looking after myself; and (v) Communicating, collaborating and connection with others (about our child and family). It is hoped that what one parent has called “early intervention for parents” will positively influence their approach to their child and family's development and functioning (16).

The ideas we are promoting are built around the F-words concepts described earlier in this paper. We want to encourage parents to take a *strengths-based approach* to child and family *development*; to help families understand that while we cannot “fix” developmental impairments we can *promote functioning*—however it is done—and enable parents to see their children as *having capability* even in the face of impairments. It is essential to *recognize the enormous diversity in human functioning*, and to move away from the tyranny of “normal” and *celebrate achievement, however it is accomplished*. Finally, we encourage people to take a *life-course approach* to their predicaments, based on their ever-changing child (and family) and the continuing evidence of what they can do if given the opportunities.

We are offering these ideas to parents in an effort to help them “reset” their thinking about themselves, their child, their control over their lives. Understanding the concepts of *capacity* and *performance* can be useful, reminding parents that what we do at our best (*capacity*) is not always how we *perform* on a day to day basis. This idea can be important if it allows parents, and us, to explore the factors associated with the gap between capacity and performance, and to recognize opportunities—often with environmental interventions, but also perhaps with counseling—to minimize this gap and maximize performance.

### That All Sounds Lovely in Theory, but…

Our work to promote understanding and uptake of the F-words for Child Development is enabling us to acquire a considerable body of evidence of the currency and power of these ideas around the world. For many parents, these ideas are transformative. I can do no better than to end this essay with the words of parents and colleagues, whose anecdotes and testimonials speak volumes about how the *content* of our ideas has impacted them and their families.

“*Thank you for making [a podcast about an editorial* (6)*] as this came in perfect timing for our family. X… (child with CP) is working on potty training by herself and the other day (we didn't know she was in the bathroom) she had a BM and tried to clean herself, but obviously it was messy and got all over the toilet. I had to take a deep breath because I wanted to yell at her for the mess, but then you popped into my mind. At one of our check-ups with you, a long time ago, you had said you cared about function rather than it being perfect. So instead of yelling at her I applauded her for the great try and that we would work on getting better.” (A parent, May 2021)*

“*Today in clinic I saw a child (who has CP and is functioning at a GMFCS V)—whose mother attended CP-NET Science and Family Day last Wednesday. She reported to me that her entire approach to raising her son has shifted from one of ‘fixing' to embracing the F-words and a wellness approach. She came to clinic with her goals related to the F words all worked out and felt very empowered! I couldn't be more pleased.” (From a colleague, May 2017)*

“*Today I saw a 6-year-old girl with CP GMFCS V, refugee from XXX via YYY, parents tried Stem cell therapy in ZZZ and came here (Canada) with hopes to help her. We had a long consult with a whole team of therapists, social work, resident and myself… trying to answer all the questions they had. I tried to explain what we understand by a functional approach and what the purpose of therapy is and that the exact etiology (nyd) probably won't change this approach. At the end, I showed them the F-words poster in Arabic, they read carefully, asked if these words are meant to be the child speaking and I confirmed. The mom commented under tears “this is beautiful, that's what I wish for my daughter.” (From a colleague, July 2019)*

In summary, our work with families of children and youth with long-term challenges of health or development can be incredibly rewarding and productive. The ICF's biopsychosocial framework for health, and the F-words ideas that bring these concepts to life for parents and families, provide a common language and approach for families and service providers to connect and share ideas. The f-word “family” reminds people that the nuclear environment of the child is potentially the most important of all, and demands attention from us regarding what we do and how we do it. By working as a team, in partnership with parents (and with children whenever possible), we can formulate shared goals, strive to empower families and young people to achieve their goals however they do so, and live lives that are rich and successful in their own terms. What we say and do, and how we do it, are under our control. I hope this paper has provided some evidence to support this assertion.

## Author Contributions

The author confirms being the sole contributor of this work and has approved it for publication.

## Conflict of Interest

The author declares that the research was conducted in the absence of any commercial or financial relationships that could be construed as a potential conflict of interest. The handling editor declared a past co-authorship with the author.

## Publisher's Note

All claims expressed in this article are solely those of the authors and do not necessarily represent those of their affiliated organizations, or those of the publisher, the editors and the reviewers. Any product that may be evaluated in this article, or claim that may be made by its manufacturer, is not guaranteed or endorsed by the publisher.

## References

[1] World Health Organization. International Classification of Functioning, Disability and Health (ICF). (2001). Geneva: World Health Organization.

[2] Rosenbaum PL Gorter JW. The “F-Words” in childhood disability: i swear this is how we should think! Child Care Health Dev. (2012) 38:457–63. 10.1111/j.1365-2214.2011.01338.x 22040377

[3] Robb-Smith AHT. Osler's changing influence. J R Coll Physicians Lond. (1993) 27:456–64. 8289171 PMC5396692

[4] Rosenbaum P King S Law M King G Evans J. Family-centred services: a conceptual framework and research review. Phys Occup Ther Ped. (1998) 18:1–20. 10.1080/J006v18n01_01

[5] Rosenbaum P. “You have textbooks; we have story books.” Disability as perceived by professionals and parents. Editorial Dev Med Child Neurol. (2021) 62:660. 10.1111/dmcn.14491 32367523

[6] Rosenbaum P. To enhance function, promote children's development. Dev Med Child Neurol. (2021) 628:14838. 10.1111/dmcn.14838 33973240

[7] Rosenbaum P. Developmental disability: shouldn't grandparents have a place at the table? Dev Med Child Neurol. (2016) 58:528. 10.1111/dmcn.13125 27171024

[8] Novak-Pavlic M Abdel Malek S Rosenbaum P Macedo LG Di Rezze B. A scoping review of the literature on grandparents of children with disabilities. Disab Rehab. (2021) 2020:1–15. 10.1080/09638288.2020.1857850 33478262

[9] Rosenbaum PD. Google versus the health practitioner: can we still deliver? Editorial Dev Med Child Neurol. (2018) 60:530. 10.1111/dmcn.13710 29740822

[10] King G Law M King S Rosenbaum P. Parents' and service providers' perceptions of the family-centredness of children's rehabilitation services in Ontario. Phys Occup Ther Ped. (1998) 18:21–40. 10.1080/J006v18n01_02

[11] King G King S Rosenbaum P Goffin R. Family-centred caregiving and well-being of parents of children with disabilities: linking process with outcome. J Ped Psychol. (1999) 24:41–52. 10.1093/jpepsy/24.1.41

[12] King S Teplicky R King G Rosenbaum P. Family-centred service: does it make difference for children with cerebral palsy or other disabilities? Sem Ped Neurol. (2004) 11:78–86. 10.1016/j.spen.2004.01.009 15132256

[13] Siegler M. Pascal's wager and the hanging of crepe. N Engl J Med. (1975) 293:853–7. 10.1056/NEJM197510232931705 1177976

[14] Rosenbaum P Stewart D. Alternative and complementary therapies for children and youth with disabilities. Infants Young Child. (2002) 15:51–9. 10.1097/00001163-200207000-00008

[15] Rosenbaum P. Complementary and alternative therapies: what are our responsibilities? Dev Med Child Neurol. (2019) 61:1352. 10.1111/dmcn.14340 31680246

[16] Rosenbaum PL Novak-Pavlic M Akhbari Ziegler S Hadders-Algra M. Role of the family. In: Hadders-Algra M, editor. Early Detection and Early Intervention in Developmental Motor Disorders – From Neuroscience to Participation in Daily Life. London: Mac Keith Press (2021). p. 173–84.

